# At the Heart of the Industrial Boom: Australian Snubfin Dolphins in the Capricorn Coast, Queensland, Need Urgent Conservation Action

**DOI:** 10.1371/journal.pone.0056729

**Published:** 2013-02-20

**Authors:** Daniele Cagnazzi, Guido J. Parra, Shane Westley, Peter L. Harrison

**Affiliations:** 1 Marine Ecology Research Centre, School of Environment, Science and Engineering, Southern Cross University, Lismore, Australia; 2 Cetacean Ecology, Behaviour and Evolution Lab, School of Biological Sciences, Flinders University, Adelaide, Australia; 3 South Australian Research and Development Institute (SARDI), Aquatic Sciences, West Beach, Australia; 4 Fitzroy Basin Association Inc. Level 1, Rockhampton, Australia; Biodiversity Insitute of Ontario - University of Guelph, Canada

## Abstract

The recent industrial boom along the Australian coastline has increased concerns about the long term conservation of snubfin dolphins along the Queensland coast. National assessment of the conservation status and management of the Australian snubfin dolphin is currently hindered by the lack of adequate biological and ecological information throughout most of its range. In response to the issue of determining the conservation status of species with broad ranges, the IUCN has provided a framework for assessing the threatened status of regional populations. In this study we assessed the conservation status of a small geographically isolated population of snubfin dolphins living in the Fitzroy River region, Queensland, Australia, against the IUCN criteria for regional populations. A review of all available sightings data and stranding information indicates that this is the southernmost resident population of snubfin dolphins in Australian waters. The Fitzroy River snubfin dolphin population is composed of less than 100 individuals, with a representative range and core area of less than 400 and 300 km^2^ respectively. The area most often used by snubfin dolphins within the representative range and core area was estimated to be about 292 and 191 km^2^, respectively. A decrease in representative range, core area and preferred habitat between 14 and 25% is projected to occur if a planned industrial port development were to occur. These results are robust to uncertainty and considering the low level of formal protection and future threats, a classification of this subpopulation under the IUCN Red List as “Endangered” is appropriate.

## Introduction

Estuaries and coastal seas have been focal points of human settlement and marine resource use throughout history [1]. Today, almost half (44%) of the world’s population lives within 150 km of a coastline, and coastal ecosystems are under unprecedented pressure. Australia’s coastline is also under increasing pressure, with an expected population increase of one million people over the next 15 years, and a predicted continuous decrease in population sizes, geographic ranges and genetic diversity of most groups of coastal biota (Australian State of the Environment 2011).

Despite their recognised ecological and outstanding international values the coastal waters within the Great Barrier Reef World Heritage Area, along the east coast of Queensland, are also affected by coastal development. Industrial ports, mining and industrial activities have been the main factor driving population growth (40% increase from 2009) throughout the Great Barrier Reef (GBR), at rates faster than the Australian average [2]. A recent IUCN report [3] highlighted that this unprecedented scale of developments throughout the GBR poses serious concerns over the long-term conservation of the entire reef.

Given the rapid growth of coastal developments and the implications of this for the conservation of coastal delphinids, there is increasing scientific and public concern about the long term survival of the Australian snubfin (*Orcaella heinsohni*, hereafter snubfin dolphin) in affected areas [4]–[6]. Four key factors underlie the risks to these small cetaceans: (1) their life history features make them particularly vulnerable to human impacts (e.g. slow life history with low reproductive rates, and high trophic levels); (2) their restricted distribution to estuarine, and/or near-shore marine habitats [7], [8]; 3) their small population sizes [9]–[11] and 4) their potential endemism to Australian waters [12], [13].

**Figure 1:**
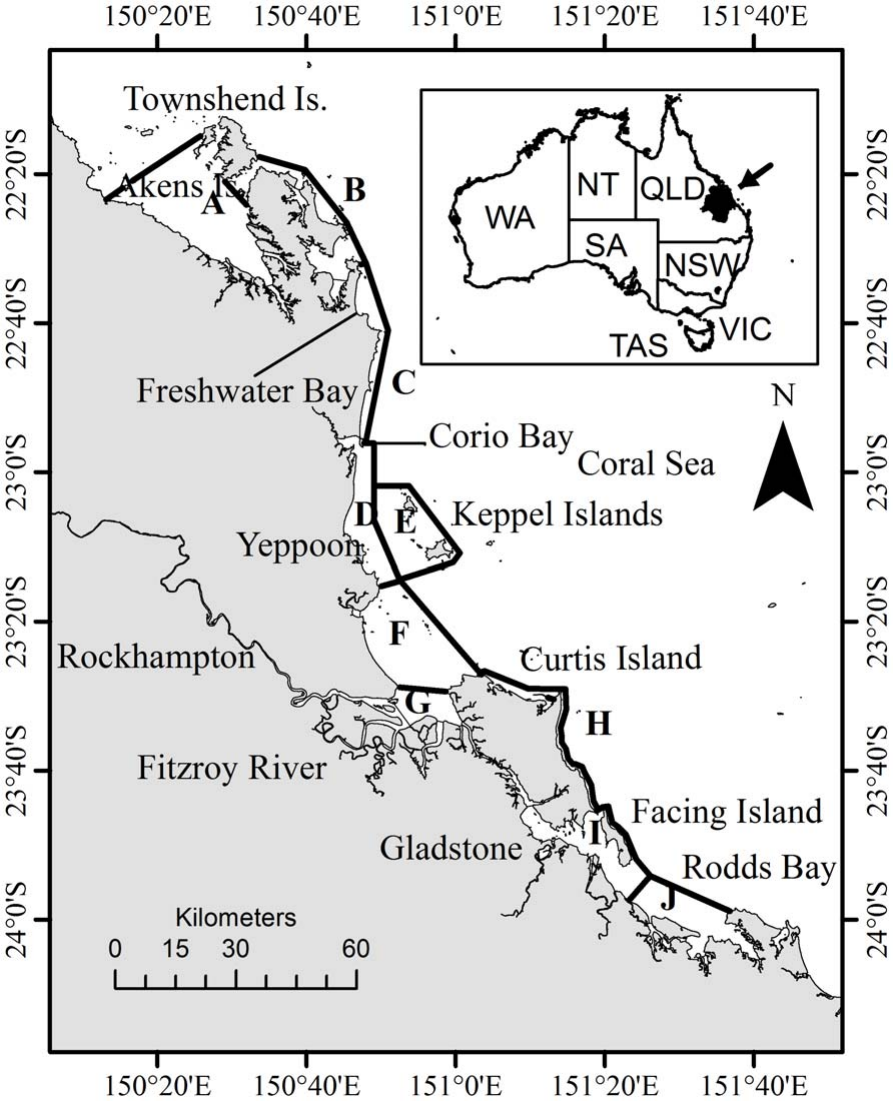
Map of the Capricorn Coast study area. Capital letters indicate survey sections: A = Shoalwater Bay, B = Northern Regions, C = Nine Miles Beach, D = Keppel Bay inshore, E = Keppel Islands, F = Fitzroy outer estuary, G = Fitzroy inner estuary and river, H = Curtis Island East Coast, I = North Port Curtis, J = South Port Curtis. Shoalwater Bay Military Training Area = A+B¸ Keppel Bay = D+E+F+G, Port Curtis = I+J.

One of the Queensland coastal areas undergoing major development is the Capricorn-Curtis Coast, within the Capricorn section of the Great Barrier Reef Marine Park (GBRMP). This section of coastline (Fig. 1), because of its natural protected waters and proximity to inland coal mines, has been the preferred site for a major industrial expansion of the Port of Gladstone that began in 2010 [14] and for the possible construction of a new industrial port in the nearby Fitzroy River [15]–[17].

After large scale marine fauna mortality events, recorded in Gladstone’s Port Curtis in 2010–2011 [18], there are increasing concerns about the potential effects that concurrent adverse weather conditions and developments proposed within the Fitzroy River estuary [3], [15], [16] may have on the local marine environment and particularly on a resident small population of snubfin dolphins.

At present, the assessment of the conservation status of Australian snubfin dolphins is hindered by the lack of adequate biological and ecological information throughout most of their range across northern Australia. At a national level, the snubfin dolphin is listed as “migratory” and “cetacean” under the Commonwealth Environment Protection and Biodiversity Conservation Act 1999 [19]. At a state and international level snubfin dolphins are listed as “near threatened” in the Queensland Nature Conservation Act 1992 (NCA) and IUCN Red List of Threatened Species 2008.

In this study, we present comprehensive information on the distribution, habitat use, abundance and potential effects of human-caused mortality of a small isolated snubfin dolphin population resident in the Fitzroy River estuary within the Capricorn Section of the Great Barrier Reef Marine Park. This information is fundamental for future re-assessments of the conservation status of snubfin dolphins in Queensland and Australia. Using data collected from this and other related studies, the southern distribution range of snubfin dolphins along the Australian east coast was also confirmed. The conservation status of this population was assessed against IUCN Red List criteria for regional populations [20].

Our results highlight the vulnerability of this population, and more broadly the need for urgent conservation actions in view of the increasing industrial development and other human activities affecting much of the east coast of Queensland and other areas of northern Australia where snubfin dolphins occur.

## Materials and Methods

### Study Area and Data Collection

The study area extends for approximately 360 km along the Central Queensland coast from Rodds Bay (151.675°E, −24.087°S) in the south, to Akens Island (150.265°E, −22.362°S) in the north, and coincides with Capricorn Section of the Great Barrier Reef Marine Park (Fig. 1). To guarantee the inclusion of the total area potentially used by snubfin dolphins in the surveys, the 25 m contour depth was selected as the general offshore limit of the study area as snubfin dolphins are rarely seen in offshore waters deeper than 20 m [8].

Within this region three principal areas were selected for dedicated boat based surveys: 1) Port Curtis, 2) Keppel Bay, and 3) Shoalwater Bay Military Training Area (Fig. 1). These areas were chosen based on known inshore dolphin habitat requirements [8], [21] and data collected during preliminary surveys conducted in 2006 throughout the entire region.

Due to their large sizes, survey areas were divided in sections (Fig. 1), and within each section, vessel surveys followed a combination of zig zag routes in open areas and strip transect surveys in creeks, small inlets and rivers. Due to the high tidal range and variable weather conditions, extensive areas may or may not be available for surveys. Therefore survey routes in open areas were decided on a daily basis depending on the weather conditions and tide level with the primary aim of covering one entire section in a day. Open coastlines (Curtis Island East Coast and Nine Miles Beach) were surveyed only during movement to and between the three surveys areas following zig-zag line transect surveys conducted between the coastline and the 25 m depth contour offshore.

Vessel-based surveys were completed between 2006 and 2011, in calm sea conditions (i.e. swell <0.8 m and Beaufort sea state <2) and at an approximate speed of 5–10 knots. During transect surveys, two or three observers searched for dolphin schools (i.e. aggregation of dolphins with relatively close spatial cohesion and involved in similar behavioural activities) with one observer on each side of the boat, and the boat driver watching ahead.

After a school of dolphins was sighted, dolphins were approached slowly to collect photo-identification data (using a Nikon D100 digital camera equipped with an 80-mm to 400-mm zoom lens), date, time, geographical location (latitude and longitude, recorded using a GARMIN GPS 178C Sounder), school size, and age composition (i.e. adults, juveniles and calves after Parra et al 2006a).

Photos taken during surveys were divided into three categories: 1) photos that included the entire dorsal fin and dorsal fin base, and whose quality (according to focus, contrast, angle to the animal, and the size of dorsal fin relative to the frame) allowed the primary and secondary marks to be distinguished; 2) photos whose quality allowed each animal in the school to be distinguished by age class, but were not suitable, due to the absence of prerequisites outlined at point (1), to distinguish individual dolphins between schools; and 3) poor quality photos. Only photos belonging to the first category were used to catalogue new individuals and to distinguish among them.

The field work was conducted under Southern Cross University Animal Care and Ethics Committee approval n° 10/16 and under scientific research permit of the Great Barrier Reef Marine Park Authority G10/33405.

### Representative Range and Core Area

The representative range and core area were estimated at the population level, using locations of schools of animals rather than of individuals’ locations. The term population was used to identify the portion of the global population found within the study area as defined within IUCN Red List for regional assessment [22].

To avoid dependence among sightings, if a school of dolphins was sighted more than once in the same day only the first record was included in the analysis. Snubfin dolphins representative range and core areas were estimated using the Kernel method at 95% and 50% utilization distributions (i.e., relative use of space) respectively, using smoothing factors calculated via least squares cross-validation because these produced the most accurate estimates [23], [24]. Kernel ranges were generated with Geospatial Modelling Environment [25] and ArcMap v 10 [26].

To estimate the proportion of snubfin dolphins habitat found within GBRMP conservation zones and potentially affected by the dredging, shipping routes, mooring sites, berths and loading areas proposed in the various port developments within the Fitzroy River estuary [3], [15], [16], the representative range and core area probability contours were overlaid over GIS shapefiles (i.e. geographically referenced files) of the GBRMP conservation zone limits [27]and over a layer representing an approximation of the dredging, shipping routes, mooring site, berth and loading areas (hereafter referred to simply as port development) proposed in the various port developments plans within the area [16], [17]. The final extent of the intersected areas was estimated using Analysis and Spatial Statistic tools in ArcMap 10. GIS shapefiles of the Marine Park borders were obtained from the Great Barrier Reef Marine Park Authority while the layer resembling the proposed development was digitized in ArcGIS 10 using publicly available maps [17].

### Habitat Selection

We used the Manly’s alpha Electivity Score (α_r_) [28] to determine the habitat preferences of snubfin dolphins in relation to water depth within their representative range and core area:
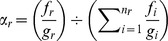
where *fr* = number or record of schools found in habitat r (with r = 1 to n_R_), and *gr* = percentage cover of habitat type r. Alpha values vary from 0, if the taxon of interest completely avoids the habitat, to 1 if there is an absolute preference for a particular habitat. If each habitat type is used in proportion to its availability, all *α_r_* are equal [29].

To define the different habitat types available according to bathymetry, a depth grid with a cell resolution of 50 x 50 m was created for the study area using the inverse distance weighting interpolation method implemented in ESRI ArcGIS 10. Spatial bathymetry data were obtained from the Fitzroy Basin Association, Marine Safety Queensland and from data collected during boat surveys. A categorical variable depth grid was then created for six depth categories (*r*): intertidal area (0 to 2 m), shallow waters (2–5 m), subtidal waters (5–10 m), moderate depth waters (10–15 m), and deeper waters (15–20 m). Depth was measured at the lowest level of astronomical tide as shown in the navigation chart. Data were projected into Universal Transverse Mercator WGS 1984 UTM Zone 56S. To determine the percentage cover of habitat type r and the number of schools seen in each habitat, the depth grid was intersected with the representative range, core area and sightings layers.

The null hypothesis that habitats were selected randomly in proportion to their availability (H_0_: 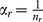), was tested against the alternative hypotheses of habitat preference or avoidance (H_a_: 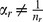), using the Bray-Curtis Index of Dissimilarities (BCD) [30] as the test statistic:
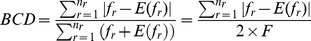



BCD varies between 0, for completely matching vectors, to 1 for the most extreme dissimilarity. Statistical significance was tested using randomisation procedures (n = 10,000), which compare the observed usage values against the expected usage values within the study area [29], [31].

Expected usage values were estimated under assumption of H_0._ Under this hypothesis the total number of schools sighted (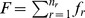) should be subdivided per habitat according to its availability, therefore the expected number of schools per habitat r was estimated as *E(f_r_)* = *F × g(r)*. Expected values were obtained by randomly assigning the total number of schools (*F*) to each habitat type *r* with a probability *g(r)* following a multinomial distribution [29]. This procedure was repeated 10,000 times using randomisation techniques and the significance of the test evaluated by counting how many times pseudo-BCD values exceeded the observed values. Resample and randomisation tests were run using POPTOOLS version 3.2 [32].

To test which of the habitats was used more or less often than expected we used the difference (*D_r_*) of *α_r_* estimate from 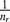 (expected distribution under H_0_) as the test statistic. P-values were determined estimating the proportion of pseudo *D_r_* values, obtained with 10,000 randomisations, further away from 0 than the *D_r_* value obtained from observed data. In this test, the tendency of dolphins to use a specific habitat more or less often than expected by chance is indicated by significant positive or negative *D_r_* values respectively. To avoid type 1 error, *P*-values were adjusted using Bonferroni correction for multiple comparisons [29].

The availability of the preferred area within the representative range and core area were estimated by erasing the layer representing habitat types used less often than expected by chance, from the 95 and 50% probability polygons using Analysis and Spatial Statistic tools in ArcGIS v 10. The total amount of preferred habitat within the GBRMP border and that affected by the proposed development was estimated following the same protocol.

### Population Estimates

To obtain estimates of the population size of snubfin dolphins within the study area, we used the Arnason’s parameterization of the Jolly–Seber open population model [33], [34] available in the computer program MARK [35]. This model provides abundance estimates while allowing entries (i.e., births, immigration) and losses (i.e., death, permanent emigration), and thus is suitable for longer-term studies. The assumptions required under the Jolly-Seber open population model are listed and validated in Table 1.

**Table 1:** Validation of the assumptions involved in Jolly-Seber capture–recapture models used for the estimation of population sizes of snubfin dolphins in Keppel Bay survey area.

Assumptions and validations	References
1) Mark recognition and mark loss	[39], [68]
(a) Only category 1 photographs of dolphins were used to identify and catalogue individuals.	
(b) Mark-Recapture analysis was limited to dolphins with long lasting marks.	
(c) Regular boat based survey allowed to monitor eventual changes in marks.	
(d) Secondary marks like scars were also used to limit the risk of misidentification.	
2) Homogeneous capture and survival probabilities.	[69]–[71]
(a) Heterogeneity in capture probabilities was reduced by collapsing all sighting efforts within a field season (March to October)to a single event (seen or not seen).	
(b) The pooled χ2 statistics (Test 2+ Test 3*) indicated that this assumption was not violated (χ2 = 8.09, df = 10, p = 0.619).	
(c) The directional test for transients was not significant (Z = 0.12, df = 4, p = 0.451*).	
(d) Capture probabilities were relatively high (see section 3.4).	
2) No behavioural responses	[72].
(a) Photo-identification is a remote and non-invasive technique, thus dolphins are not subject to stress or risks involved in ‘capture’ orphysical marking.	
(b) Pradel’s Test for trap dependence of marked individuals was not significant (Z = 0, df = 3, p = 1*).	
3) Instantaneous sampling: sampling occasions selected for analysis were relatively short in duration (8 months) in comparison with thedolphins’ lifespan (decades)	[69]
4) Permanent and temporary emigration	[11], [68], [71]
(a) Estimates of recapture probabilities were relatively high (see section 3.4).	
(b) No indication of heterogeneity in capture and survival probability (Test 2+ Test 3^a^).	
(c) About 400 km of coastline were surveyed during this study including regions to the south and north of the present population.	
(d) The Fitzroy river population is geographically isolated.	
(e) Preliminary genetic results indicate that this population is genetically isolated.	

* The pooled χ^2^ test statistic (Test 2+Test 3) the directional test for Transients and the Pradel’s Tests were carried out using the program U-Care [73].

At first the full parameterised model with a total of six capture occasions (*p*), five survival (*φ*) and immigration probabilities (*Pent*) estimates, and one population estimate for marked individuals (*N_m_*) were fitted to the data. Estimates for each parameter were obtained using the Pam-specific option, capture and survival probabilities were estimated using the sin function, the immigration probabilities with the MLogit function, while marked population estimates with the Log function.

Then, to increase accuracy in the parameter estimates, a set of reduced models was built. The models were adjusted to account for data overdispersion by incorporating the inflation factor (ĉ) into the modelling and inference methods to better reflect the uncertainty in precision. The inflation factor was calculated in MARK changing data-type in “live recaptures” using the median approach [36]. Final model rank was based on the quasi-likelihood modification of Aikake Information Criterion, QAICc, [37]. The total population size over the entire study (N_total_) and per sampling period (N_p_) was estimated by taking into account the proportion of marked individuals over the entire study period (*θ_total_*) and within sampling seasons (*θ_p_*). The proportion of marked individuals was estimated from the number of individuals identified from category 1 photographs showing a recognisable individual. Standard errors for the total population size were derived from its variance (Var) given by:
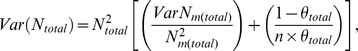
where *n* is the total number of animals from which *θ* was estimated [38], [39]. Standard errors for the yearly population estimates were derived with the same formula replacing *N* and *θ* values with the corresponding values for each capture occasion.

### Potential Biological Removal

To investigate the potential effect of human-caused mortality in the study area the Potential Biological Removal (PBR) approach was used [40]. PBR is an estimate of the maximum number of animals, not including natural mortalities, which may be removed from a marine mammal stock while allowing that stock to reach or maintain its Optimum Sustainable Population [40]. PBR was calculated as:
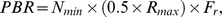
where *N_min_* is the 20th percentile of the population size estimate, *R_max_* the maximum annual population growth rate and F_r_ the recovery factor. *N_min_* estimates and standard errors were estimated from this study as



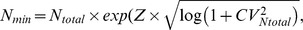
where CV is the coefficient of variation and *Z* is the standard normal deviate corresponding to a specific percentile, fixed at 0.842 for the 20^th^ percentile [40]. To account for variability in population estimates *N_total_* values were randomly drawn from a normal distribution with mean and standard deviation generated in this study using the variable number creator in POPTOOL 3.2 [32].

As no direct estimates of population growth rates are available for snubfin dolphins, R_max_ value was set at 0.04, considered to be a standard value for cetaceans [40]. Final PBR values were estimated setting F_r_ at values expected for endangered species (Fr = 0.1), species at an intermediate or unknown conservation status (F_r_ = 0.3, F_r_ = 0.5), and for species not at risk (F_r_ = 1) [41]. Average PBR values and errors estimates were calculated running 5,000 Monte Carlo simulations in POPTOOLS v. 3.2 [32].

## Results

### Survey Effort and Photo-identification

Between 2006 and 2011, a total of 3 847 hrs of survey time were completed during surveys from March to October: 1 421 hrs were spent surveying Keppel Bay; 1 168 hrs surveying Port Curtis, 989 hrs in Shoalwater Bay Military Training Area, 174 along Nine Miles Beach and 95 hrs along East Curtis Coast. A total of 568 schools of snubfin dolphins were sighted, from which 77 adult individuals were identified. Of the 568 schools of snubfin dolphins sighted throughout the study area, 566 were recorded within the Keppel Bay study area, one in Shoalwater Bay and one along the East Curtis Coast sections. On 31 occasions, photo-identification was unsuccessful; therefore data from these sightings were not used in further analysis.

**Figure 2:**
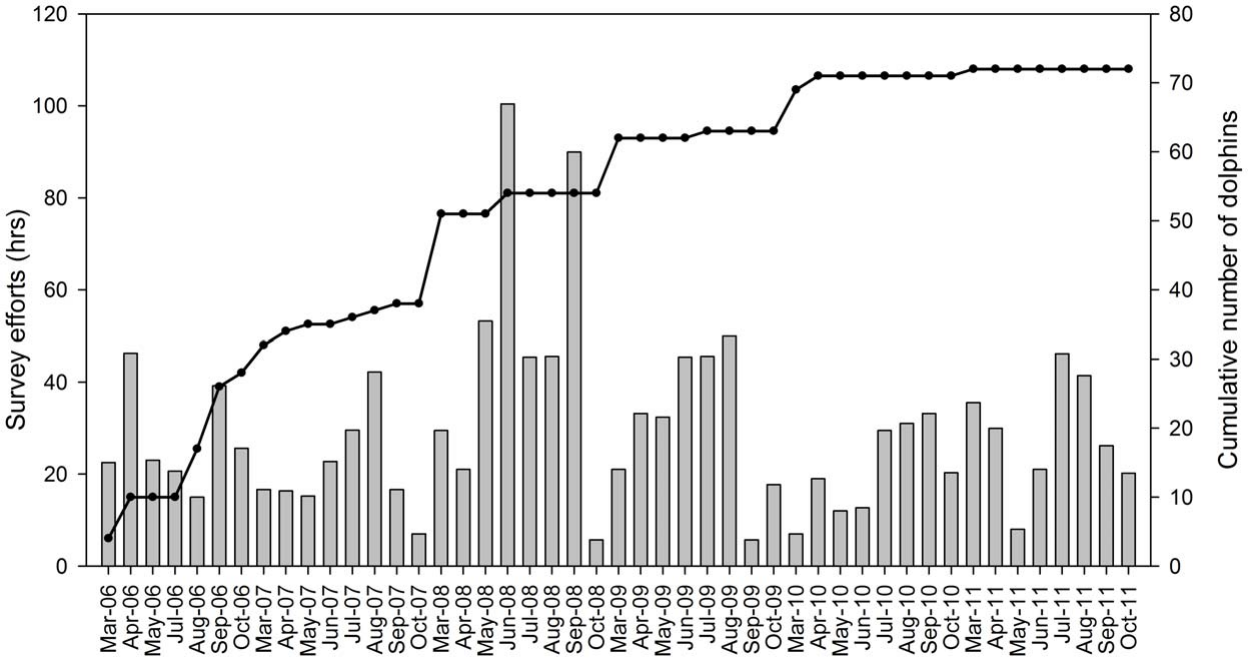
Discovery curve of marked snubfin dolphins. Discovery curve showing the cumulative number of snubfin dolphins (grey line) identified in relation to hours of survey effort per month (grey bars) in Keppel Bay between 2006 and 2011.

As a result of the lack of snubfin dolphin sightings in the Shoalwater Bay Military Training Area, Nine Miles Beach, East Curtis Coast and Port Curtis, we limited all analyses on ranging, habitat utilization, population estimates and potential biological removal to the Keppel Bay study area. The discovery curve (i.e. cumulative number of identified animals; Fig. 2) increased constantly until mid-2010 when a plateau was reached; suggesting that the population was open for the duration of most of the study and/or that unrecognisable animals acquired new recognisable marks as the study progressed.

Overall sighting frequency was high with 64 dolphins, out of the 77 identified, sighted in four or more field seasons, while only four dolphins were sighted in a single sampling season (mean = 4.63, SE = 0.52). Within each sampling season snubfin dolphins were re-sighted frequently with an average of 0.57 sighting/month (SE = 0.04).

### Representative Ranges and Core Areas

**Figure 3:**
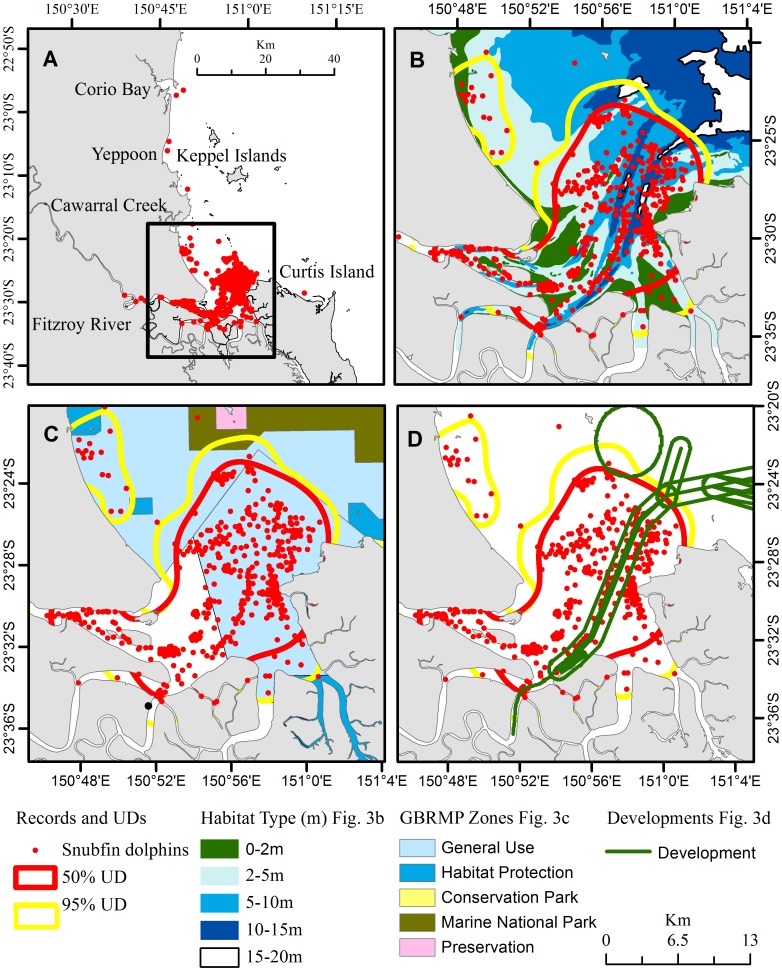
Limited protection for snubfin dolphins in an increasingly developed environment. Map of the Keppel Bay survey area showing snubfin records with red circles (**A**), of the representative range (yellow line) and core areas (red line) in relation to the habitat type (**B**), to Great Barrier Reef Marine Park zones (**C**), and to the proposed development (dredging areas, shipping routes, mooring sites, berth and loading areas) in the Fitzroy River estuary (**D**). Scale bar is located on the bottom right of the figure unless specified in the map.

Within Keppel Bay, the number of sightings decreased moving farther away from the river, with only seven records in the Keppel Bay inshore section and none in the Keppel Island section (Fig. 3A). The resulting representative range (95% kernel UD) of snubfin dolphins is formed by a main area coinciding with the Fitzroy River estuary and one smaller area in the proximity of Cawarrall Creek (Fig. 3B). Photo-identification data showed that animals move between these two areas, indicating that only a single population is present in the area. The core area (50% kernel UD) was formed by a single area coinciding with the Fitzroy River estuary. The representative range and core area of space use by snubfin dolphins were estimated to be approximately 349 and 231 km^2^, respectively. Only 88 km^2^ of the representative range and 13 km^2^ of the core area were within the GBRMP borders, of which, 70% (79 km^2^), and 100% (13 km^2^) respectively were found in a GBRMP General Use Zone (Fig. 3C). The study area that is projected to be directly modified by the proposed port development overlaps with 14% (49 km^2^) of the representative range and 17.7% (41 km^2^) of the core area used by snubfin dolphins (Fig. 3D).

### Habitat Availability

The Bray-Curtis Index of dissimilarities (BCD) showed significant differences between habitat use and availability within overall and core ranges (95% UD: BCD_obs_ = 0.13, BCD_exp_ = 0.03, 95% CI = 0.01–0.05, P = 0; 50%UD: BCD_obs_ = 0.15, BCD_exp_ = 0.07, 95% CI = 0.04–01, P = 0). The *Dr* tests (Table 2) for selection or avoidance of each habitat showed that snubfin dolphins used shallow water (2–5 m), shallow subtidal water (5–10 m) and moderate depth water (10–15 m) habitats within their representative ranges and core areas more frequently than expected by chance. In contrast, intertidal (0–2 m) and deeper water (15–20 m) habitats were used less frequently than expected by chance.

**Table 2:** Habitat use availability analysis for snubfin dolphins using the Manly’s alpha Electivity Score and corresponding p-values.

Habitat type	UD	Area	g(r)	n	α_r_	Dr_obs_	E(D_r_) (95% CI)	p
	%	(km^2^)						
Intertidal area	95	57.4	0.17	28	0.07	−0.12	0.03 (0.01–0.05)	0
(0–2 m)	50	39.9	0.17	17	0.05	−0.15	0.04 (0.02–0.06)	0
Shallow Interior	95	153.5	0.46	274	0.25	0.05	0.01 (−0.03–0.03)	0
(2–5 m)	50	94.8	0.41	252	0.31	0.11	0.03 (0.01–0.08)	0
Shallow Subtidal	95	83.9	0.25	170	0.29	0.09	0.01 (−0.03–0.04)	0
(5–10 m)	50	68.4	0.30	165	0.28	0.08	0.02 (0.00–0.04)	0
Moderate depth	95	30.2	0.09	67	0.32	0.12	0.00 (−0.04–0.05)	0
(10–15 m)	50	22.9	0.10	65	0.33	0.13	0.00 (0.00–0.05)	0
Deeper water	95	5.6	0.01	2	0.05	−0.14	−0.002 (−0.1–0.1)	0
(15–20 m)	50	5.6	0.02	2	0.04	−0.16	−0.05 (−0.12–0.03)	0

In the table the following notation was used: Habitat Type = depth range in m at lowest astronomical tide, UD% = utilisation distribution at 95% kernel UD for representative range and 50% kernel UD for core area, Area = area of habitat available, g(r) = probability of observing a school of snubfin dolphins in a certain habitat type; n = number of dolphin schools detected; a_r_ = Manly’s alpha index; Dr_obs_ = observed deviation between a_r_ and 1/n_R_; E(Dr) = expected deviation between a_r_ and 1/n_R_.

Excluding the intertidal and deeper water habitats, the total area most often used by snubfin dolphins within the representative range and core area, was estimated to be about 292 and 191 km^2^ respectively, of which 17 and 25% is projected to be modified by the proposed development (Fig. 3).

### Model Selection and Population Estimates

The variation inflation factor ĉ was less than 2 (Mean = 1.4, SE = 0.07), indicating limited over dispersion in the data; nevertheless the models were adjusted accordingly. The full time dependent model, although had the lowest QAICc value, provided inaccurate estimates for all parameters (Table 3). The model with good data fit and accurate estimates for all parameter was reached by setting survival and entry probabilities to be constant throughout the study and capture probabilities to vary with time.

**Table 3:** List of Jolly-Seber models that reached numerical convergence and associated population estimates per sampling period.

Model	QAICc	np	Year	n	N_m(p)_		SE	CI	θ_p_	N_p_	CI
*φ*(t)*p*(.)*pent*(.)*N* a	267.8	8	2006	31		59	6.4	47–72	0.74	80	68–93
*φ*(.)*p*(t2)*pent*(.)*N*	268.7	7	2007	41		58	4.7	49–67	0.78	74	65–84
*φ*(.)*p*(t)*pent*(.)*N*	272.8	9	2008	54		57	3.7	50–64	0.78	73	66–80
*φ*(.)*p*(.)*pent*(.)*N*	275.0	4	2009	53		56	3.4	49–62	0.76	73	66–80
*φ*(*t*)*p*(.)*pent*(*t*)*N*	3 0706	12	2010	53		55	3.9	47–62	0.75	73	65–80
*φ*(.)*p*(.)*pent*(*t*)*N*	3 0715	8	2011	52		54	4.7	45–63	0.76	71	61–80
*φ(t)p(t)pent(t)N* b	259.9	17	All	77		81	2.5	78–89	0.77	105	100–110

The models are ranked based on the quasi-likelihood modification of the Akaike information criterion (QAICc). Marked and total population estimates are derived from the model with good fit of the data and accurate estimates for all parameter (second in the table).

In the table the following notation was used: *φ* = survival probability; *p* = capture probability; *pent* = probability of entry in the population; *N* = population estimates; np = number of parameters; *t* = time dependent effect; and • = constant effect; n = number of dolphins marked per sampling season; *N_m(p)_* = marked population estimates per sampling period; N_p_ = total population estimates per sampling season; θ_p_ = proportion of marked individuals per sampling period; CI = 95% confidence intervals; SE = standard error. N_p_ = N_m(p)_/θ_p_.

a The first model listed in the table generated inaccurate estimates for two survival parameters.

b The full time dependent model generated inaccurate estimates for all Beta parameters.

Overall capture probabilities were high throughout the study (*p_2006_* = 0.55, SE = 0.09; *p_2007_* = 0.66, SE = 0.08; *p_2008, 2009, 2010_* = 0.86, SE = 0.03; *p_2011_* = 0.92, SE = 0.05). Adult survival rate also was high (*φ* = 0.9, SE = 0.02, 95% CI = 0.84**–**0.94) and within the range of those usually recorded for other long-lived cetacean species [42], [43]. Immigration probability was very low (*Pent* = 0.05, SE = 0.01, 95% CI = 0.02**–**0.09) as expected for geographically isolated populations formed mainly by resident individuals. Abundance estimates from 2006 to 2011 indicate that about 70 to 80 individuals used the study area each year, and that about 100 individuals used this area over the six years of this study (Table 3).

### Potential Biological Removal

The range of PBR estimates for sustainable anthropogenic mortality are summarised in Table 4 for the 2006 and 2011 population estimates of snubfin dolphins. Based on these values, regardless of the recovery factor used, the sustainable anthropogenic mortality estimate remained well below 2 individuals per annum (Table 4).

**Table 4:** Estimates of the potential biological removal (PBR) of Australian snubfin dolphins for the 2006**–**2011 abundance estimates obtained in the Keppel Bay region for four distinct recovery factors (F_r_ = 0.1, 0.3, 0.5, 1).

Years	N_min_	CV	PBR_(Fr = 0.1)_	PBR_(Fr = 0.3)_	PBR_(Fr = 0.5)_	PBR_(Fr = 1)_
			(95%CI)	(95%CI)	(95%CI)	(95%CI)
2006	74.8	0.08	0.15 (0.12–0.17)	0.44 (0.37–0.51)	0.74 (0.63–0.86)	1.5 (1.2–1.7)
2007	71.6	0.06	0.14 (0.12–0.16)	0.43 (0.37–0.48)	0.71 (0.62–0.80)	1.4 (1.2–1.6)
2008	71.1	0.05	0.14 (0.13–0.16)	0.42 (0.38–0.47)	0.71 (0.63–0.77)	1.4 (1.3–1.6)
2009	71.4	0.05	0.14 (0.13–0.16)	0.43 (0.39–0.47)	0.71 (0.64–0.78)	1.4 (1.3–1.6)
2010	70.4	0.05	0.14 (0.12–0.15)	0.42 (0.37–0.47)	0.70 (0.63–0.78)	1.4 (1.3–1.6)
2011	67.7	0.07	0.14 (0.12–0.15)	0.41(0.35–0.46)	0.67 (0.58–0.76)	1.3 (1.2–1.5)
TOT.	104	0.03	0.21 (0.19–0.21)	0.62 (0.59–0.65)	1.0 (0.9–1.1)	2.1 (1.9–2.2)

The table also shows the average estimates of 20th Percentile of Population Size (*N_min_*) and associated coefficient of variation (CV).

In the Table PBR = *N_min_* ×(0.5×*R_max_*)×*F_r_*. The maximum annual population growth rate (R_max_) was set at 0.04 [40].

## Discussion

### Overall Distribution

In this study a total area of about 4,000 km^2^ of coastal habitats was surveyed, including regions to the north, the south and east of the Fitzroy River estuary. Our results indicate that snubfin dolphins in the Capricorn coast are not homogeneously distributed, but instead these dolphins were encountered primarily (with only one exception) in the Keppel Bay survey area. Within this region, the representative range of the population extended over 349 km^2^ and was composed of two areas; one large area coinciding with the Fitzroy River estuary and a smaller area in the proximity of Cawarrall Creek. The core area of use coincided with the Fitzroy River estuary, which further highlights the importance of this estuary system for the long term survival of this population of snubfin dolphins.

The size of the representative range and core area recorded in this study were substantially larger than those recorded for snubfin dolphins in Cleveland Bay; 197 and 43 km^2^, respectively [21]. The habitat preferences of snubfin dolphins in Keppel Bay also partly differ from those in Cleveland Bay where snubfin dolphins showed a preference for waters 0 to 2 m in depth [21]. In contrast, in the Keppel Bay study area snubfin dolphins showed a preference for waters between 2 and 15 metres in depth which may indicate some level of local adaptation as expected in species with isolated populations with restricted ranges. However, further detailed studies are needed to further investigate the relationships between snubfin dolphin habitat preferences and environmental, biological and physical variables.

Edge populations, are particularly important for the long-term conservation of genetic diversity, phylogenetic history and evolutionary potential of a species [44]. In addition to the information presented in this study, a series of vessel-based line transect surveys were conducted between May and September 2008 from Cape Palmerstone (21.53°S, 149.47°E) to Mackay (21.12°S, 149.22°E) and Mackay to the Whitsunday Islands region (20.07°S, 148.56°E). These surveys indicated that snubfin dolphins were frequently seen only in Repulse Bay (20.63°S, 148.72°E) at the southern end of the Whitsundays (Parra and Cagnazzi unpublished data), which is located about 500 km to the north of the Keppel Bay survey area in this study. South of the Fitzroy River, the occurrence of snubfin dolphins is considered extralimital [7], [45], as no record of snubfin dolphins has been reported south of the Keppel Bay study area from long term studies on inshore dolphins in the Great Sandy Strait, Hervey Bay [9] and in Moreton Bay further south [46]. Furthermore, only four stranding records have been documented in the Queensland Wildlife Marine Stranding and Mortality Database (1998**–**2007) south of the Fitzroy River. This information indicates that the Fitzroy River population is not only geographically isolated from population of conspecifics along the Queensland coast, but also that the Fitzroy River snubfin dolphins represent the southernmost resident population of snubfin dolphins along the Australia’s east coast. Therefore, the recognised southern boundary of Australian snubfin dolphin populations along the east Australian coast should be the Fitzroy River, not the Brisbane River. As a population at the edge of their geographic their investigation and conservation deserve high priority [44].

### Population Size and Potential Biological Removal

We consider the population estimates presented in this study to be accurate and unbiased because 1) field and data analysis procedures were chosen as to minimize biases, and 2) violations of the assumptions of Jolly-Seber population models were not detected. Furthermore, the entire area used by the local population including surrounding regions outside the population range was surveyed consistently throughout the year for the duration of the study.

Estimates of snubfin dolphin population size remained relatively stable throughout the study, indicating that less than 90 snubfin dolphins use the Fitzroy River on a yearly basis. These estimates are very close to the population estimate for the entire period, as expected in a closed population with limited or no immigration from surrounding regions. Considering the high resighting pattern of marked individuals, the absence of nearby populations of snubfin dolphins to the north and south of the Keppel Bay study area, and the stability of yearly population estimates, significant immigration from or emigration to neighbouring populations appears unlikely. However, there is likely to be occasional movement outside the described range, as suggested by the single record of snubfin dolphins in Shoalwater Bay. Therefore, the population estimates for snubfin dolphins in the entire study area and possibly north to Mackay, could be derived from the overall population estimates obtained in this study, which indicates that about one hundred individuals live in the Central Queensland coastal region.

The small population size in Keppel Bay, coupled with its geographic isolation, highlights the vulnerability of snubfin dolphins in this region. Despite the various scenarios considered and the conservative approach applied to determining the total sustainable anthropogenic mortality (Potential Biological Removal), the results indicate that the loss of more than one dolphin per year as a result of human impacts is not sustainable.

### Implications for Conservation

The need for effective conservation plans for small cetaceans is clearly illustrated by examples of critically endangered or endangered cetacean species and subpopulations around the world; the Vaquita (*Phocoena sinus*) in Gulf of California, the Hector’s dolphin (*Cephalorhynchus hectori*) in New Zealand, the Indo-Pacific humpback dolphin (*Sousa chinensis*) in Taiwan and Hong Kong, and the Irrawaddy dolphin (*Orcaella brevirostris*) in Asia [47**]**–[**51**]. Moreover, the most cautionary example is that of the recent (functional) extinction of the Yangtze River dolphin (*Lipotes vexillifer*) [52], which represents the first known cetacean extinction in recent history.

Although the Fitzroy River snubfin dolphin population is not currently under threat, anthropogenic pressures and consequently conservation concerns may increase in the near future. If approved, the proposed developments within the Fitzroy River estuary will directly modify between 14 to 25% of the dolphins’ habitat, without accounting for indirect effects of development activities on the nearby habitat and the long term consequences of daily operating activities on the entire area. Only small proportions of the representative range (25%) and core area (5%) are within the GBRMP borders, of which 78% and 100% are within a GBRMP General Use Zone. The General Use Zone offers the lowest level of protection among the various GBRMP conservation zones allowing a wide range of uses such as fishing, aquaculture, shipping and boating. Thus, the level of protection for the Fitzroy River snubfin dolphins’ population within the GBRMP is very limited.

A positive correlation exists between non-random habitat loss and extinction rates [53]. The minimum amount of habitat that needs to be preserved to allow persistence of all species in a region varies, however species with low reproductive potential and a low dispersal strategy, such as snubfin dolphins, require very large amounts of habitat for persistence. Furthermore, the carrying capacity of a population is directly related to the productivity and quality of its home range (Macdonald & Rushton 2003, Mitchell & Powell 2004). If the size of the home range is decreased, or restricted during certain periods, it will decrease the productivity available to the population, which in turn could decrease the population size (Singer et al. 2001, Mitchell & Powell 2004). To remain stable, the population would have to increase its home range in order to make up for lost resources, or alternatively the population size may decrease. Considering the geographic isolation of this population and that its existence and survival appear to be related entirely to the quality and quantity of habitat provided by the Fitzroy River estuary, and that no other similar habitat occurs within or in proximity to the study area, an increase in the home range size appears unlikely. Furthermore, a decrease in population size will increase the effects of random genetic processes that can lead to a reduction of the effective population size, and a reduction in the genetic variability and the evolutionary potential [54]. Thus conservation of habitat quality within the Fitzroy River estuary will be critical for the long-term survival of local population of snubfin dolphins.

The Fitzroy River snubfin dolphin population is small, comprising of less than 100 individuals with an average potential biological removal of less than 1 dolphin per annum. Given that in species with slow life history the response to habitat loss is delayed [55], we are not likely to be able to detect a problem with this population until well after the habitat or its population size has been reduced below their threshold. Therefore, a precautionary approach to the management of this population is required.

### Assessment Under the IUCN Red List Criteria for Regional Populations

The IUCN has provided a framework for assessing the threatened status of subpopulations (IUCN 2001), defined as geographically or otherwise distinct groups in the population, between which there is little demographic or genetic exchange (typically one successful migrant individual or gamete per year or less). Using this framework, five Irrawaddy dolphin (*Orcaella brevirostris*) subpopulations of the Mahakam River, Malampaya Sound, Mekong River, Songkhla Lake, Ayeyarwady River [56**]**–[**59**] and the eastern Taiwan Strait humpback dolphin [48] subpopulation were recently classified as “Critically Endangered”.

Analysis of the distribution of sightings indicates that the Fitzroy River population of Australian snubfin dolphins is geographically isolated from conspecific populations outside the region. This hypothesis is supported by preliminary genetic analysis [11]. Therefore, considering that 1) the taxon reproduces within the region, 2) the likelihood of immigration from nearby population/s is low, 3) there is evidence of local adaptation, and that 4) this species along the Queensland coast is relatively uncommon and live in populations of small size, the Fitzroy River snubfin dolphin population should be considered as a distinct subpopulation as per the IUCN definition [20]. Under this scenario, the IUCN Red List Criteria can be used for the assessment of regional populations without modification and a regional population may be classified as critically endangered (CR), endangered (EN) or vulnerable (VU), if any one of five criteria and associated sub-criteria are met: (A) reduction in population size, (B) geographic range, (C) population size and presence of a decline, (D) population size, or (E) probability of extinction. In this study we were able to assess the Fitzroy River snubfin dolphins populations against the criteria B and D (Table 5).

**Table 5:** IUCN Red List criteria met by the Fitzroy River snubfin dolphin population and the resulting threat classifications: vulnerable (VU), endangered (EN) or critically endangered (CR) (IUCN, 2001).

Criteria	Sub Criteria	Classification
B1(a)	MCP = 1 255 km^2^	EN<5000 km^2^
B2(b)	95% UD = 349 km^2^ 50% UD = 231 km^2^	EN<500 km^2^
(1)(c)	1) The Fitzroy River snubfin dolphin population is geographically isolated with a representative rangerestricted to the Fitzroy River estuary.	x
2ii, 3iii(c)	1) 14% of the representative range and 17% of the habitat preferred by snubfin dolphin is projected to be modified by the port development.	x
	2) A continued decline in habitat quantity and quality is expected during the contruction and operationalphases.	
3ii. 3iii(c)	1) Summer floods richer in contaminants and sediments are expected to cause major fluctuationin the area of occupancy and quality of the habitat.	x
	2) Extreme fluctuations are expected during construction phases	
D(d)	N_(total)mature_ = 52 (50–55) N_(p)mature_ = 40 (31–46)^e^	CE<50 EN<250
	N_(total)mature_ = 63 (60–67) N_(p)mature_ = 48 (37–55.8)^f^	

(a) B1 =  extent of occurrence. The extent of occurrence was estimated using the Minimum Convex Polygon as indicated in the 2001 IUCN Red List Categories and Criteria version 3.1. No sub-criteria are required.

(b) B2 =  area of occupancy. The representative range at 95% UD was used as a proxy of area of occupancy.

(c) The classification under Criteria B1 and B2 requires 2 between the following sub-criteria: 1) severely fragmented or known to exist at only a single location and 2) continuing decline, observed, inferred or projected and 3) extreme fluctuations in any of the following: (i) extent of occurrence (ii) area of occupancy (iii) area, extent and/or quality of habitat (iv) number of locations or subpopulations (v) number of mature individuals.

(d) D = Mature Population size calculated at 50% (^e^) and 60% (^f^) of the total population size (N_total_) and of the population size per sampling period (N_p_) as estimated for humpback and bottlenose dolphins [62]. No sub-criteria are required for Criteria D.

The criteria B specifies threshold values for extent of occurrence (i.e. the area contained within the shortest continuous imaginary boundary which can be drawn to encompass all the known sites of present occurrence of a population, excluding cases of vagrancy) and area of occupancy (i.e. the area within its ‘extent of occurrence’ which is occupied by the population, excluding cases of vagrancy) (Table 5) [22]. For the purpose of this assessment we presented an estimate for the extent of occurrence using the Minimum Convex Polygon (MCP) as indicated in the 2001 IUCN Red List Categories and Criteria version 3.1, although we note that there are bias in using MCP to estimate extent of occurrence [60]. While, as no specific method was suggested to estimate the area of occupancy, the representative range at 95%UD was used as a proxy of the area of occupancy. Both the extent of occurrence and area of occupancy are well within the threshold required under the IUCN criteria to assign an “Endangered” status to this population (Table 5). The Fitzroy River estuary may soon undergo substantial habitat modification if several projects associated with the coal export are approved. As a result, a substantial decline in the area of occupancy is projected, while extreme fluctuations in habitat quantity and quality are expected during the construction stages (Table 5), as has been noted for other coastal developments [61].

The Fitzroy River snubfin dolphin population was estimated to contain substantially fewer than 250 mature individuals, and therefore can be classified as “Endangered” under criterion D. This classification is robust to uncertainty in estimates of abundance and the proportion of mature individuals in the population, given that the 95% upper confidence intervals for the yearly and absolute abundance estimates that included all individuals did not exceed 83 and 111, respectively. However, if we assume that the proportion of mature individuals may be similar to that of the Indo-Pacific humpback dolphin (*Sousa chinensis*), which is about 50%, and bottlenose dolphins (*Tursiops aduncus*) which is about 60% [62], this population would be very close to meeting the criterion for a Critically Endangered status (<50 individuals) (Table 5).

Conversely, if the results of more detailed genetic analyses indicate that conspecific populations outside the region will affect the regional extinction risk, the regional Red List Category could be downgraded to the next lower conservation status (IUCN 2003), whereby the Fitzroy River snubfin dolphin population would be at least classified as “Vulnerable”. However, considering 1) the ecological importance of this population, being the southernmost population of potentially endemic snubfin dolphins in Australia, 2) that the decline or extirpation of regional populations of top level predators such as coastal dolphins could have significant ecological effects on ecosystem structure and function [63], [64], 3) that under the IUCN criteria, the mature population size is considerably smaller than the threshold for “Endangered” and very close to the limit required for a “Critically Endangered” status, 4) the high level of anthropogenic disturbance that this population may face in the near future, 5) the low potential for biological removal, and 6) the low level of formal protection existing in the region, a classification under the IUCN of this population as “Endangered” is appropriate.

### Conclusions

Marine mammals are significant values in the Great Barrier Reef World Heritage Area in ecological, cultural and economic contexts [65]. The Australian snubfin dolphins is arguably one of the most iconic cetacean species within the Great Barrier Reef waters, and it is considered a priority species for management [66]. Nevertheless at the present their conservation status cannot be assessed due to the lack of adequate biological and ecological information.

In this study we provided further evidence suggesting that population estimates for the Great Barrier Reef are likely to be in the order of lower thousands rather than tens of thousands [67]. Considering their ecological value as top predators, their small population size, limited geographic distribution, proximity to detrimental human activities and likely future increase in anthropogenic pressure throughout the Great Barrier Reef World Heritage Area there is a strong need to increase the conservation and management of snubfin dolphins. This is especially true for the Fitzroy River snubfin dolphins population is currently under minimal protection and qualifies for the regional IUCN listing of “Endangered”.
